# Multimodal MRI Characterization of Structural and Resting-State Functional Brain Alterations in Patients with Low Back Pain: A Retrospective Study

**DOI:** 10.3390/diagnostics16162546

**Published:** 2026-08-12

**Authors:** Xiaopei Sun, Yazhou Lin, Jing Zhang, Weihuan Fang, Zhe Chen, Peng Cao, Yuehuan Zheng

**Affiliations:** 1 Department of Orthopedics, Ruijin Hospital, Shanghai Jiao Tong University School of Medicine, Shanghai 200025, China; sarapennysun@sjtu.edu.cn (X.S.); lyz12454@rjh.com.cn (Y.L.); cz12165@rjh.com.cn (Z.C.); 2 Department of Rehabilitation, Shanghai Ruijin Rehabilitation Hospital, Shanghai 200023, China; 3 Department of Radiology, Ruijin Hospital, Shanghai Jiao Tong University School of Medicine, Shanghai 200025, China

**Keywords:** low back pain, magnetic resonance imaging, neuroimaging, voxel-based morphometry, resting-state functional MRI, thalamus, functional connectivity

## Abstract

**Background/Objectives:** Low back pain (LBP) is associated with central alterations, but convergence across structural, functional, and network measures remains unclear. We characterized multimodal differences. **Methods:** Resting-state fMRI included 69 participants (37 patients, 32 controls); VBM retained 23 patients and 32 controls after structural-image quality control. Structural, local functional, and thalamic-connectivity measures were assessed. Motion control used realignment-based exclusion and Friston-24 nuisance regression. Mean Power framewise displacement was additionally calculated for all participants and compared between groups, and VBM-retained and excluded patients were compared. Models adjusted for age and sex, plus total intracranial volume for VBM. Directional maps underwent one-tailed Gaussian random field correction (voxel *p* < 0.001; cluster *p* < 0.05), without cross-direction or cross-family correction. Extracted-value and imaging–clinical analyses were exploratory. **Results:** Mean framewise displacement did not differ between groups. Patients showed lower gray matter volume in thalamic–hippocampal regions and right cerebellar lobule VIII and lower local functional measures in thalamic, orbitofrontal–striatal, and right temporal regions. Thalamic connectivity with sensorimotor, parietal, supplementary motor, and middle cingulate regions was higher. Nominal analyses showed positive left thalamic connectivity cluster 1–VAS and negative left cluster 1–JOA and right clusters 1/2–JOA; none survived false-discovery-rate correction. **Conclusions:** Multimodal MRI delineated lower thalamic structural and local functional measures alongside higher thalamo-sensorimotor and thalamo-parietal connectivity. Nominal, directionally coherent clinical associations remain hypothesis-generating. This thalamus-centered pattern warrants replication in prospectively matched, clinically stratified cohorts.

## 1. Introduction

Low back pain (LBP) remains one of the leading causes of disability worldwide, and its societal burden continues to grow [1,2,3,4]. Clinical assessment understandably focuses on lumbar pathology, such as disc degeneration, spinal stenosis, or vertebral fracture [5,6]. These findings, however, often correspond poorly with the patient’s experience. Similar lumbar images may accompany very different levels of pain, disability, and restriction in daily life.

This clinical mismatch has encouraged a broader view of LBP that includes central pain modulation, sensitization, and experience-dependent plasticity [7,8,9,10]. Persistent pain is now understood to emerge from several interacting processes rather than from peripheral nociceptive input alone. Spinal processing, thalamocortical signaling, attention, memory, affect, and sensorimotor control may all contribute [7,8,9,10]. Brain imaging offers one way to examine these central contributions alongside lumbar anatomy.

Structural and resting-state MRI capture different aspects of this process. Voxel-based morphometry (VBM) estimates regional gray matter volume. Resting-state fMRI, in turn, can describe the magnitude of spontaneous fluctuations, the local similarity of neighboring signals, and coupling between distant regions. In this setting, fractional amplitude of low-frequency fluctuations (fALFF), regional homogeneity (ReHo), and seed-based functional connectivity provide related but non-equivalent measures [11,12,13,14,15,16,17]. Considering them together may reveal changes that would be missed by a single imaging metric.

The thalamus is of particular interest because it sits at the intersection of ascending somatosensory input and distributed cortical systems. Its reciprocal connections with sensorimotor, parietal, prefrontal, cingulate, and limbic regions allow it to participate in sensory filtering, attention, arousal, and pain-related behavior [18,19,20]. Work in LBP has already pointed to altered thalamo-sensorimotor pathways and abnormal coupling with somatomotor and frontoparietal networks [21,22,23,24,25,26,27]. What is less clear is whether the same thalamic involvement can be seen across structure, local resting-state activity, regional synchrony, and large-scale connectivity.

**Figure 1:**
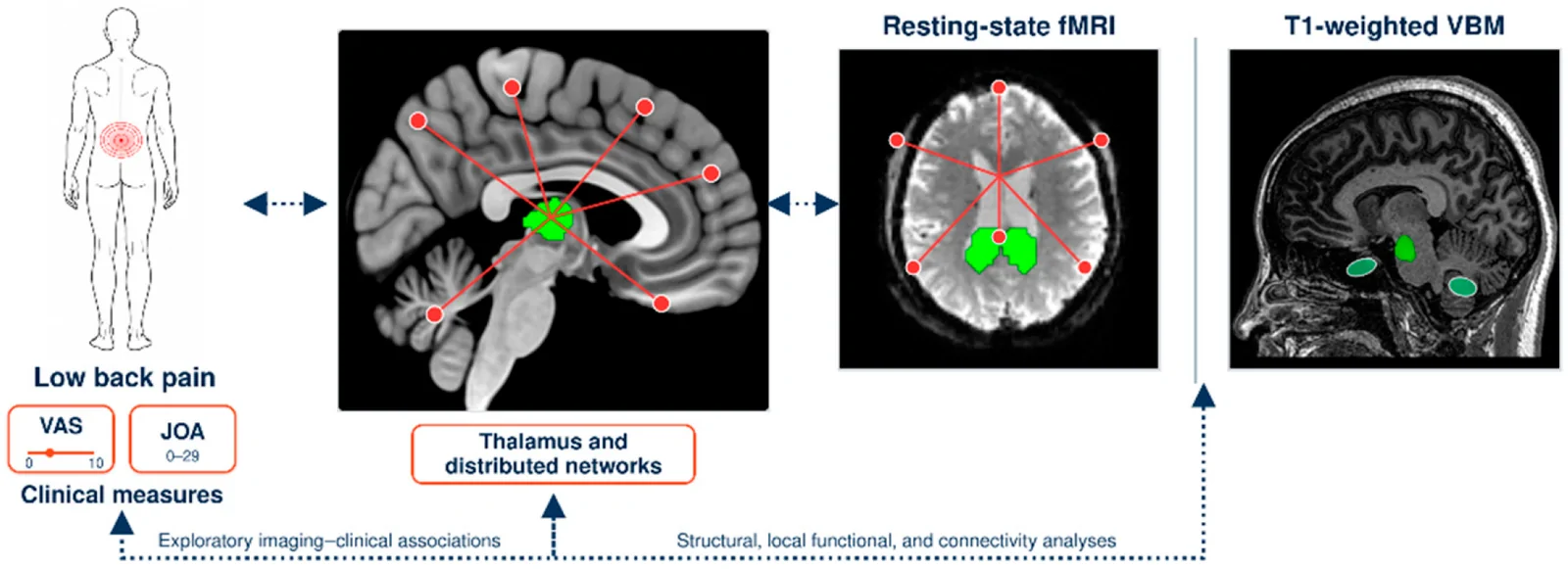
Conceptual overview of the multimodal MRI study. The study examined structural and resting-state functional brain alterations in patients with low back pain. Voxel-based morphometry was used to assess gray matter volume, while multi-metric resting-state fMRI characterized spontaneous activity, local coherence, and bilateral thalamic functional connectivity. Extracted imaging measures were subsequently examined in relation to VAS and JOA scores in exploratory patient-only analyses. Abbreviations: JOA, Japanese Orthopaedic Association; VAS, Visual Analog Scale.

We therefore combined T1-weighted VBM with several resting-state fMRI measures in patients whose LBP was attributed to clinically diagnosed lumbar disease. We also explored whether the extracted imaging measures varied with pain intensity or lumbar function, assessed using the Visual Analog Scale (VAS) and Japanese Orthopaedic Association (JOA) score. We expected thalamic structural and local functional abnormalities to occur alongside altered thalamo-sensorimotor connectivity (Figure 1). The primary objective was to characterize group-level structural and resting-state functional MRI differences between patients with LBP and healthy controls. The secondary objective was to evaluate whether the extracted effects remained detectable after demographic adjustment and whether they showed exploratory associations with pain intensity and lumbar function.

## 2. Materials and Methods

### 2.1. Study Design and Participants

This retrospective study used existing brain resting-state fMRI and T1-weighted MRI data acquired at the Department of Orthopedics, Ruijin Hospital, Shanghai Jiao Tong University School of Medicine, between October 2023 and October 2025. The Institutional Review Board of Ruijin Hospital approved the study (No. 20240902113233506) and waived written informed consent. No additional recruitment, intervention, or imaging was performed for this analysis. Because this was a retrospective analysis of all eligible imaging records available during the predefined study period, no prospective sample-size calculation was performed.

A total of 37 patients with low back pain (PTs) and 32 healthy controls (HCs) were included. In patients, LBP was attributed to a clinically diagnosed lumbar disorder, including degenerative disc disease, lumbar fracture, or spinal stenosis. Diagnosis was based on concordant lumbar MRI findings, symptoms, physical signs, and the treating physician’s medical-record diagnosis. According to the recorded primary diagnosis, 20 of the 37 patients (54.1%) had spinal stenosis, 12 (32.4%) had degenerative disc disease, and 5 (13.5%) had lumbar fracture. Two patients with lumbar fracture had subacute LBP lasting slightly more than 1 month, whereas the remaining 35 patients, including the other three with lumbar fracture, had chronic LBP lasting more than 3 months. Medication exposure and physical-therapy history were not consistently available; diagnosis-stratified analyses were therefore not performed. For descriptive reporting, each patient was assigned to one primary diagnostic category.

Healthy controls had no history of LBP or other chronic pain and no major neurological or psychiatric disorder. Available clinical assessments and brain T1-weighted images showed no clinically significant abnormality.

Exclusion criteria for both groups were previous spinal surgery, major neurological or psychiatric disease, claustrophobia, unstable systemic illness, incomplete clinical records, or insufficient imaging data.

Pain intensity was assessed using the VAS score recorded closest to the MRI examination. Lumbar function was assessed with the JOA score.

### 2.2. MRI Acquisition

MRI data were acquired using a 3.0-T GE SIGNA Architect IQE MRI system (GE HealthCare) at the Department of Radiology, Ruijin Hospital, Shanghai Jiao Tong University School of Medicine. During resting-state fMRI, participants lay supine with their eyes closed and were instructed to remain awake, minimize head movement, and avoid deliberate cognitive activity.

Resting-state functional MRI data were acquired using a gradient-echo echo-planar imaging (GRE-EPI) sequence with interleaved axial slice acquisition (slice order: 1:2:43, followed by 2:2:42). The acquisition parameters were as follows: 43 slices; repetition time/echo time (TR/TE), 2000/30 ms; flip angle, 90°; field of view, 220 × 220 mm^2^; acquisition matrix, 64 × 64; slice thickness, 3.2 mm; interslice gap, 0 mm; voxel size, 3.4 × 3.4 × 3.2 mm^3^; 240 volumes; number of excitations, 1; and parallel imaging acceleration factor, 2. No dummy scans were acquired by the scanner. The total acquisition time was 8 min.

High-resolution T1-weighted anatomical images were acquired in the sagittal plane using a three-dimensional spoiled gradient-recalled echo (3D-SPGR) sequence. The acquisition parameters were as follows: repetition time/echo time (TR/TE), 8.1/3.1 ms; inversion time, 450 ms; flip angle, 8°; field of view, 256 × 256 mm^2^; acquisition matrix, 256 × 256; slice thickness, 1 mm; interslice gap, 0 mm; isotropic voxel size, 1 × 1 × 1 mm^3^; receiver bandwidth, 31.25 kHz; and parallel imaging acceleration factor, 2. A total of 180 sagittal slices were acquired, including two oversampling slices at each end; after removal of these four slices, 176 slices were retained. The total acquisition time was 5 min 1 s.

Although no dummy scans were acquired prospectively, the first 10 functional volumes were discarded during preprocessing to allow for signal stabilization.

### 2.3. Resting-State fMRI Preprocessing, Data Calculation, Statistical Analysis, and Visualization

Resting-state fMRI data were preprocessed in MATLAB R2022b using RESTplus v1.31_20250123 and associated REST functions [11,12]. DICOM images were converted to Neuroimaging Informatics Technology Initiative format [13], and the first 10 volumes were discarded for signal stabilization. Preprocessing included slice-timing correction and rigid-body realignment. Participants were excluded if maximum absolute translation exceeded 3.0 mm or maximum absolute rotation exceeded 3.0°; all 69 participants in the final functional dataset passed these criteria. Functional images were normalized to MNI152 space using T1-based normalization, resampled to 3 × 3 × 3 mm^3^, visually inspected for registration accuracy, and spatially smoothed as specified for each metric. Nuisance regression included the Friston-24 motion model and mean white-matter and cerebrospinal-fluid signals; global signal regression was not performed. After linear detrending, temporal filtering was applied according to the metric-specific processing branches described below. Separately from preprocessing, mean Power FD was calculated from the six rigid-body realignment parameters across the 230 retained volumes for every participant and analyzed as a supplementary group-level motion assessment (Supplementary Table S1). Mean FD was not used for volume censoring or as a second-level imaging covariate. Cardiac and respiratory recordings were unavailable; therefore, preprocessing was expected to reduce, but not eliminate, physiological and other non-neuronal contributions.

RESTplus was used to calculate fALFF, ReHo, and seed-to-voxel functional connectivity [14,15,16,17]. We analyzed mean-scaled and z-standardized fALFF (mfALFF and zfALFF) and smoothed mean-scaled and z-standardized Kendall’s coefficient of concordance ReHo maps (smKCC-ReHo and szKCC-ReHo). After slice-timing correction, realignment, spatial normalization, nuisance regression, and linear detrending, separate processing branches were used for the different resting-state measures. fALFF was calculated from the unfiltered time series as the ratio of power within 0.01–0.08 Hz to that across the full detectable frequency range. ReHo was calculated from unsmoothed data using Kendall’s coefficient of concordance and was subsequently spatially smoothed with a 6 mm full-width at half-maximum Gaussian kernel. For seed-based functional connectivity, the preprocessed time series were band-pass filtered at 0.01–0.08 Hz before seed-to-voxel correlation analysis. The left and right thalamic seed masks were defined using the bilateral thalamic regions of the Automated Anatomical Labeling atlas (AAL116). The masks were resampled to the functional image resolution in MNI space. For each participant, the mean time series was extracted separately from each thalamic seed and correlated with the time series of every voxel within the whole-brain analysis mask. Pearson correlation coefficients were transformed into z values using Fisher’s r-to-z transformation before group-level analysis.

Group comparisons were performed in SPM12 using two-sample *t*-tests with age and sex as covariates of no interest. The HC > PT and PT > HC directions were evaluated as separate one-tailed contrasts, and each map underwent Gaussian random field (GRF) correction at voxel *p* < 0.001 and cluster *p* < 0.05 [28,29]. No additional correction was applied across the two directions or across the fALFF, ReHo, and seed-connectivity analysis families; these whole-brain results are therefore interpreted as exploratory. Mean-scaled and z-standardized maps represent alternative normalizations of the same underlying metric and are not independent replications. Statistical maps were inspected and visualized using xjView (version 9.6), MRIcroGL (version 1.2.20220720), and BrainNet Viewer (version 1.63) [30,31]. Detailed fMRI results are provided in Supplementary Tables S4–S9.

### 2.4. T1-Weighted Structural Preprocessing, VBM Calculation, Statistical Analysis, and Visualization

High-resolution T1-weighted images were used for voxel-based morphometry (VBM) because their 1 × 1 × 1 mm^3^ isotropic spatial resolution provided substantially finer anatomical detail than the resting-state functional images. T1-weighted images were processed in MATLAB R2022b using the Computational Anatomy Toolbox (CAT12.9, revision 2577; released 3 May 2024) implemented in Statistical Parametric Mapping software (SPM12, revision 7771) [32,33]. Each participant’s T1-weighted image was visually inspected and cropped when necessary before preprocessing. Using the default CAT12 volume-based processing pipeline, the images were corrected for intensity inhomogeneity and segmented in native space into gray matter, white matter, and cerebrospinal fluid. The gray-matter segments were then spatially normalized to standard Montreal Neurological Institute space and modulated using the corresponding deformation fields to preserve local tissue volume. Surface reconstruction and region-of-interest analyses were disabled because the present study focused on whole-brain voxel-based gray-matter volume. The resulting modulated, spatially normalized gray-matter maps (mwp1 images) were used for subsequent analyses.

Image quality was assessed using the CAT12 quality-control reports. Only participants with a CAT12-derived image quality rating (IQR) of ≥80% were retained. After quality control, the VBM dataset comprised 32 healthy controls and 23 patients with low back pain. Total intracranial volume (TIV) was calculated within CAT12 as the sum of gray-matter, white-matter, and cerebrospinal-fluid volumes. The modulated normalized gray-matter maps were smoothed using an 8 mm full-width at half-maximum Gaussian kernel, producing smwp1 images for statistical analysis.

To assess potential selection associated with structural-image quality control, patients retained in the VBM analysis (*n* = 23) were compared with excluded patients (*n* = 14) with respect to CAT12 image-quality rating, age, sex, VAS, JOA, TIV, and total gray-matter volume. Age and VAS were compared using Mann–Whitney U tests, sex using Fisher’s exact test, and JOA, TIV, and total gray-matter volume using Welch independent-samples *t*-tests. Hedges’ g and 95% confidence intervals were reported where applicable. TIV and gray-matter estimates derived from images below the quality threshold were treated as exploratory because segmentation error could affect those measurements (Supplementary Table S2).

Whole-brain between-group differences in gray-matter volume were examined in SPM12 using a two-sample *t*-test within the general linear model. Group was entered as the variable of interest, while age, sex, and TIV were included as covariates of no interest. An absolute gray-matter masking threshold of 0.20 was applied; no additional explicit mask was used. The PT > HC and HC > PT directions were evaluated as separate one-tailed contrasts, and each map underwent GRF correction at voxel *p* < 0.001 and cluster *p* < 0.05. No additional correction was applied across directions or across imaging-analysis families; the results are therefore exploratory. Significant maps were visualized using xjView and MRIcroGL. Detailed VBM results are provided in Supplementary Table S3.

### 2.5. Baseline Demographic and Clinical Statistics

Baseline demographic and clinical analyses were performed in MATLAB R2022b. The normality of continuous variables was assessed using the Lilliefors test. Normally distributed continuous variables were compared using independent-samples *t*-tests and are reported as mean ± standard deviation. Sex distributions were compared using the chi-square test. All tests were two-sided, and *p* < 0.05 was considered statistically significant.

As a supplementary assessment of between-group head motion, mean Power framewise displacement was analyzed for all 69 participants in the functional dataset. Because mean FD was non-normally distributed, the primary comparison used the Mann–Whitney U test and is reported as median and interquartile range. Mean ± standard deviation, the Welch independent-samples *t*-test, the PT−HC mean difference with its 95% confidence interval, and Cohen’s d are additionally reported for comparability and effect-size estimation. A linear model further assessed the mean FD group difference after adjustment for age and sex.

### 2.6. Methods for Selection-Dependent Post Hoc Analyses of Extracted Values

Given the marked between-group differences in age and sex, selection-dependent post hoc analyses were performed using mean values extracted from the significant imaging clusters. For the two VBM clusters, separate ANCOVA models included group, age, sex, and TIV. For the nine local resting-state fMRI clusters, separate models included group, age, and sex; Benjamini–Hochberg FDR correction was applied across the nine group coefficients. Because the clusters were defined from the same participants and contrasts, these analyses are circular with respect to cluster selection and cannot independently validate the whole-brain findings or remove demographic confounding.

As a second descriptive analysis, age- and sex-related effects were removed from each extracted local fMRI measure and the residualized values were compared between groups, with FDR correction across the nine comparisons. These analyses show only how the selected cluster estimates behave under alternative covariate treatments. They cannot recreate a prospectively matched sample or establish LBP-specific effects.

### 2.7. Imaging-Value Extraction and Clinical Correlation Analyses

Mean values were extracted from each significant between-group imaging cluster. For VBM, the imaging–clinical analyses included total whole-brain gray-matter volume and mean gray-matter values extracted from each significant VBM cluster. Imaging–clinical analyses were restricted to patients because VAS and JOA scores had no variability in controls. Partial correlations assessed associations with VAS and JOA, adjusting for age, sex, and TIV for VBM and for age and sex for fMRI. Tests were two-sided. Benjamini–Hochberg FDR correction was applied jointly across all 36 imaging–clinical tests in Supplementary Table S11. Uncorrected *p* values are retained for transparency, but only FDR q < 0.05 was considered statistically significant; nominal *p* < 0.05 findings are labeled exploratory.

Imaging-value extraction, partial correlations, and scatter plots were performed in MATLAB R2022b; the correlation heat map was generated in GraphPad Prism (version 8.0.2; GraphPad Software, San Diego, CA, USA).

### 2.8. Use of Generative AI

During manuscript preparation and revision, ChatGPT (web-based service, accessed from June to August 2026; OpenAI, San Francisco, CA, USA) was used to assist with English-language polishing and editorial restructuring. It was not used for study design, data collection, image preprocessing, statistical analysis, or generation of the original study data or figures. All AI-assisted text was critically reviewed and edited by the authors, who independently verified the scientific content against the original analyses and assume full responsibility for the manuscript.

## 3. Results

### 3.1. Participant Characteristics

Following functional-image quality control, all participants were retained in the resting-state fMRI dataset, comprising 32 healthy controls and 37 patients with LBP (Table 1A). All 69 participants included in the final functional dataset passed the prespecified head-motion exclusion criteria of maximum absolute translation ≤3.0 mm and maximum absolute rotation ≤3.0°. In the separate supplementary motion assessment, mean Power FD did not differ significantly between groups. Median mean FD was 0.112 mm (IQR, 0.085–0.184 mm) in healthy controls and 0.155 mm (IQR, 0.084–0.225 mm) in patients (Mann–Whitney U = 674.0, *p* = 0.327). Corresponding mean ± SD values were 0.148 ± 0.094 and 0.178 ± 0.121 mm. The unadjusted PT−HC mean difference was 0.030 mm (95% CI, −0.022 to 0.081 mm; Welch t = 1.160, *p* = 0.250; Cohen’s d = 0.275). After adjustment for age and sex, the group difference remained nonsignificant (β = −0.006 mm, SE = 0.033, t = −0.192, *p* = 0.849; 95% CI, −0.073 to 0.060 mm; Supplementary Table S1).

For the VBM analysis, only participants with a CAT12 image-quality rating (IQR) of ≥80% were retained, resulting in a final dataset of 32 healthy controls and 23 patients with LBP (Table 1B).

All 23 patients retained for VBM met the CAT12 image-quality threshold of ≥80%, whereas all 14 excluded patients had ratings below 80%; no healthy control was excluded. The median age was 67.0 years in retained patients and 74.0 years in excluded patients (*p* = 0.067). Mean JOA scores were 15.78 ± 3.74 and 13.93 ± 4.38, respectively (*p* = 0.200). No statistically significant differences were found in sex, VAS score, TIV, or total gray-matter volume (all *p* > 0.05; Supplementary Table S2).

In the fMRI dataset, the patient group included 37 participants and the healthy control group included 32 participants. The mean age was 67.41 ± 14.07 years in patients and 46.84 ± 13.25 years in healthy controls (*p* < 0.001). Male participants accounted for 19 of 37 patients (51.4%) and 8 of 32 healthy controls (25.0%; *p* = 0.025). The mean VAS score was 5.95 ± 1.33 in patients and 0.00 ± 0.00 in healthy controls (*p* < 0.001). The mean JOA score was 15.08 ± 4.04 in patients and 29.00 ± 0.00 in healthy controls (*p* < 0.001).

In the VBM dataset, the patient group included 23 participants and the healthy control group included 32 participants. The mean age was 65.30 ± 13.08 years in patients and 46.84 ± 13.25 years in healthy controls (*p* < 0.001). Male participants accounted for 12 of 23 patients (52.2%) and 8 of 32 healthy controls (25.0%; *p* = 0.039). The mean total intracranial volume was 1383.55 ± 111.22 mL in patients and 1436.12 ± 110.89 mL in healthy controls (*p* = 0.089). The mean VAS score was 5.91 ± 1.24 in patients and 0.00 ± 0.00 in healthy controls (*p* < 0.001). The mean JOA score was 15.78 ± 3.74 in patients and 29.00 ± 0.00 in healthy controls (*p* < 0.001).

### 3.2. Structural Changes: VBM Findings

**Figure 2:**
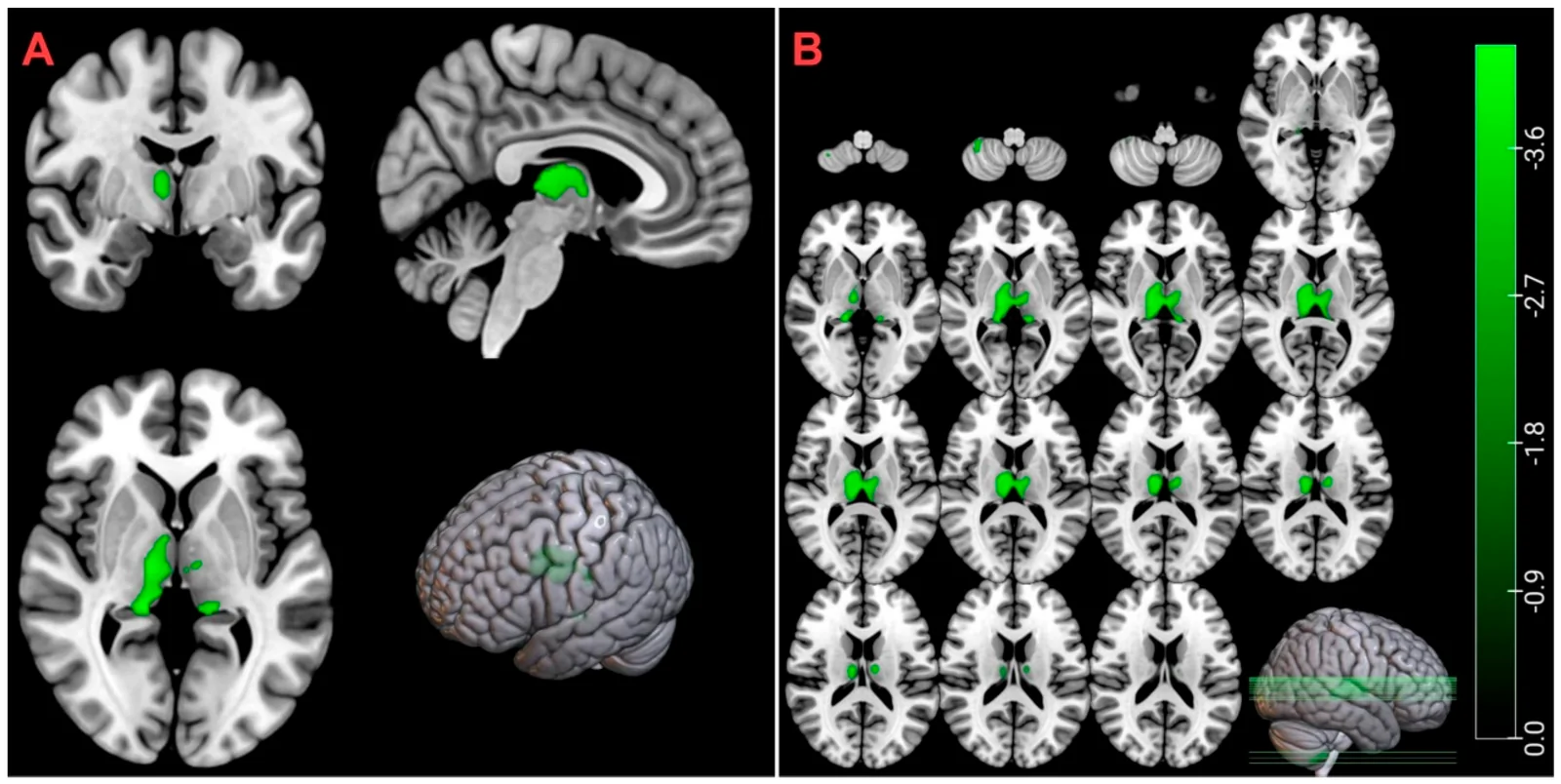
VBM-derived gray matter volume differences between PTs and HCs. (**A**) Representative coronal, sagittal, and axial views of the principal cluster showing lower GMV in PTs than in HCs, mainly involving the bilateral thalamus and hippocampus. (**B**) Multislice visualization of two clusters showing lower GMV in PTs than in HCs. Cluster 1 involved the bilateral thalamus and hippocampus (peak MNI coordinates: 13.5, −34.5, 1.5; extent: 2115 voxels; peak *t* = −4.29). Cluster 2 was located in right cerebellar lobule VIII (33, −42, −52.5; 122 voxels; peak *t* = −3.74). Green clusters indicate HCs > PTs, corresponding to lower GMV in patients. Results were corrected using one-tailed GRF correction (voxel-level *p* < 0.001; cluster-level *p* < 0.05), with age, sex, and TIV included as covariates. Abbreviations: GMV, gray matter volume; GRF, Gaussian random field; HCs, healthy controls; MNI, Montreal Neurological Institute; PTs, patients with low back pain; TIV, total intracranial volume; VBM, voxel-based morphometry.

VBM identified two decreased gray matter volume clusters in the patient group. The larger cluster comprised 2115 voxels and peaked at MNI coordinates (13.5, −34.5, 1.5), with a peak t value of −4.29. This cluster predominantly involved the bilateral thalamus, including 1253 voxels in the right thalamus and 573 voxels in the left thalamus, with additional involvement of the bilateral hippocampi. The second cluster comprised 122 voxels and peaked at MNI coordinates (33, −42, −52.5), with a peak t value of −3.74. This cluster was localized to the right cerebellar lobule VIII. These structural findings are shown in Figure 2 and detailed in Supplementary Table S3.

### 3.3. Local Spontaneous Activity: fALFF Findings

#### 3.3.1. mfALFF

**Figure 3:**
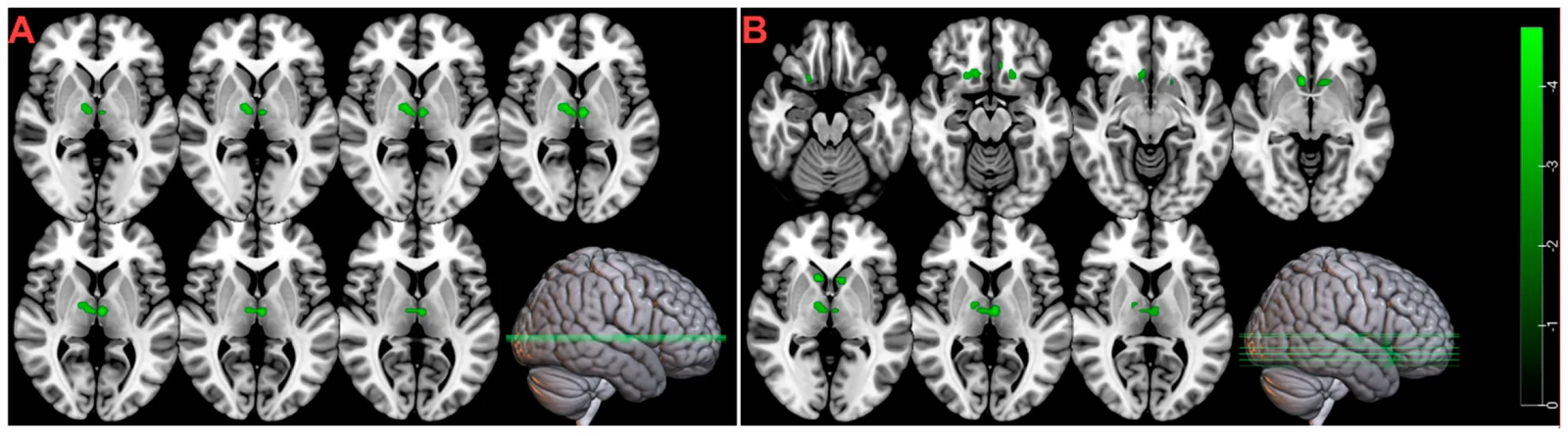
Group differences in fALFF metrics between PTs and HCs. (**A**) mfALFF analysis identified one cluster with lower values in PTs than in HCs, involving the bilateral thalamus (peak MNI coordinates: −6, −12, 3; extent: 35 voxels; peak *t* = −4.48). (**B**) zfALFF analysis identified three clusters with lower values in PTs than in HCs. Cluster 1 involved the right caudate, superior orbitofrontal gyrus, rectus gyrus, and olfactory cortex (6, 15, −3; 52 voxels; peak *t* = −4.84). Cluster 2 involved the bilateral thalamus (−6, −12, 3; 45 voxels; peak *t* = −4.63). Cluster 3 involved the left caudate, putamen, and rectus gyrus (−9, 15, 0; 32 voxels; peak *t* = −4.35). Green clusters indicate HCs > PTs, corresponding to lower fALFF values in patients. Results were corrected using one-tailed GRF correction (voxel-level *p* < 0.001; cluster-level *p* < 0.05), with age and sex included as covariates. Abbreviations: fALFF, fractional amplitude of low-frequency fluctuations; GRF, Gaussian random field; HCs, healthy controls; mfALFF, mean-scaled fALFF; MNI, Montreal Neurological Institute; PTs, patients with low back pain; zfALFF, z-standardized fALFF.

The mfALFF identified one decreased cluster in the patient group. The cluster comprised 35 voxels and peaked at MNI coordinates (−6, −12, 3), with a peak t value of −4.48. It involved the bilateral thalamus, including 20 voxels in the right thalamus and 14 voxels in the left thalamus. The spatial distribution is shown in Figure 3A and detailed in Supplementary Table S4.

#### 3.3.2. zfALFF

The zfALFF identified three decreased clusters in the patient group. The first cluster comprised 52 voxels and peaked at MNI coordinates (6, 15, −3), with a peak t value of −4.84. This cluster involved the right caudate nucleus, right superior orbitofrontal gyrus, right rectus gyrus, and right olfactory cortex.

The second cluster comprised 45 voxels and peaked at MNI coordinates (−6, −12, 3), with a peak t value of −4.63. This cluster involved the bilateral thalamus, including 25 voxels in the right thalamus and 17 voxels in the left thalamus.

The third cluster comprised 32 voxels and peaked at MNI coordinates (−9, 15, 0), with a peak t value of −4.35. This cluster involved the left caudate nucleus, left putamen, and left rectus gyrus. The spatial distribution of these clusters is shown in Figure 3B and detailed in Supplementary Table S5.

### 3.4. Local Functional Coherence: ReHo Findings

#### 3.4.1. smKCC-ReHo

The smKCC-ReHo analysis identified two decreased clusters in the patient group. The first cluster comprised 200 voxels and peaked at MNI coordinates (6, 15, −6), with a peak t value of −4.76. This cluster involved the right rectus gyrus, right olfactory cortex, and right caudate nucleus.

**Figure 4:**
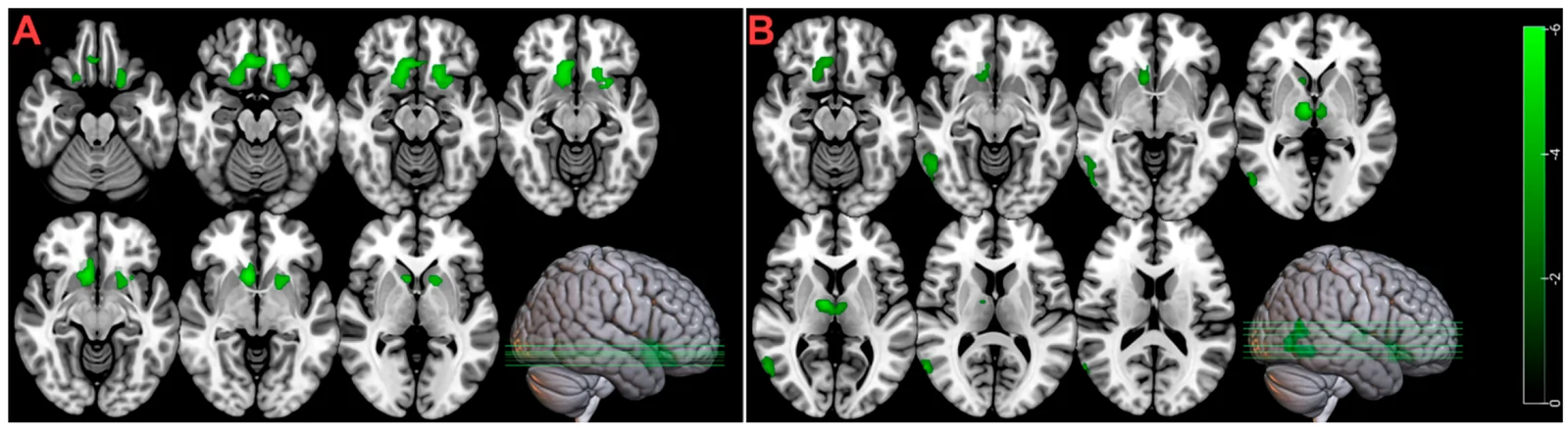
Group differences in ReHo metrics between PTs and HCs. (**A**) smKCC-ReHo analysis identified two clusters with lower values in PTs than in HCs. Cluster 1 involved the right rectus gyrus, olfactory cortex, and caudate (peak MNI coordinates: 6, 15, −6; extent: 200 voxels; peak *t* = −4.76). Cluster 2 involved the left inferior orbitofrontal gyrus and putamen (−21, 18, −18; 143 voxels; peak *t* = −4.30). (**B**) szKCC-ReHo analysis identified three clusters with lower values in PTs than in HCs. Cluster 1 involved the right inferior and middle temporal gyri (60, −63, 9; 117 voxels; peak *t* = −4.54). Cluster 2 involved the right rectus gyrus and caudate (9, 15, −3; 98 voxels; peak t = −4.41). Cluster 3 involved the bilateral thalamus (9, −9, 3; 87 voxels; peak *t* = −6.07). Green clusters indicate HCs > PTs, corresponding to lower ReHo values in patients. Results were corrected using one-tailed GRF correction (voxel-level *p* < 0.001; cluster-level *p* < 0.05), with age and sex included as covariates. Abbreviations: GRF, Gaussian random field; HCs, healthy controls; MNI, Montreal Neurological Institute; PTs, patients with low back pain; ReHo, regional homogeneity; smKCC-ReHo, smoothed mean-scaled Kendall’s coefficient of concordance-based ReHo; szKCC-ReHo, smoothed z-standardized Kendall’s coefficient of concordance-based ReHo.

The second cluster comprised 143 voxels and peaked at MNI coordinates (−21, 18, −18), with a peak t value of −4.30. This cluster involved the left inferior orbitofrontal gyrus and left putamen. The spatial distribution of these clusters is shown in Figure 4A and detailed in Supplementary Table S6.

#### 3.4.2. szKCC-ReHo

The szKCC-ReHo analysis identified three decreased clusters in the patient group. The first cluster comprised 117 voxels and peaked at MNI coordinates (60, −63, 9), with a peak t value of −4.54. This cluster involved the right inferior and middle temporal gyri.

The second cluster comprised 98 voxels and peaked at MNI coordinates (9, 15, −3), with a peak t value of −4.41. This cluster involved the right rectus gyrus and right caudate nucleus.

The third cluster comprised 87 voxels and peaked at MNI coordinates (9, −9, 3), with a peak t value of −6.07. This cluster involved the bilateral thalamus, including 51 voxels in the right thalamus and 30 voxels in the left thalamus. The spatial distribution of these clusters is shown in Figure 4B and detailed in Supplementary Table S7.

### 3.5. Thalamic Seed-Based Functional Connectivity

#### 3.5.1. Left Thalamic Seed

Voxel-wise functional connectivity analysis using the left thalamus as the seed identified three increased connectivity clusters in the patient group. The first cluster comprised 366 voxels and peaked at MNI coordinates (−45, −18, 57), with a peak t value of 4.96. This cluster involved the left postcentral gyrus, left precentral gyrus, left inferior parietal lobule, and left supramarginal gyrus.

The second cluster comprised 198 voxels and peaked at MNI coordinates (27, −21, 57), with a peak t value of 4.43. This cluster involved the right precentral and postcentral gyri.

**Figure 5:**
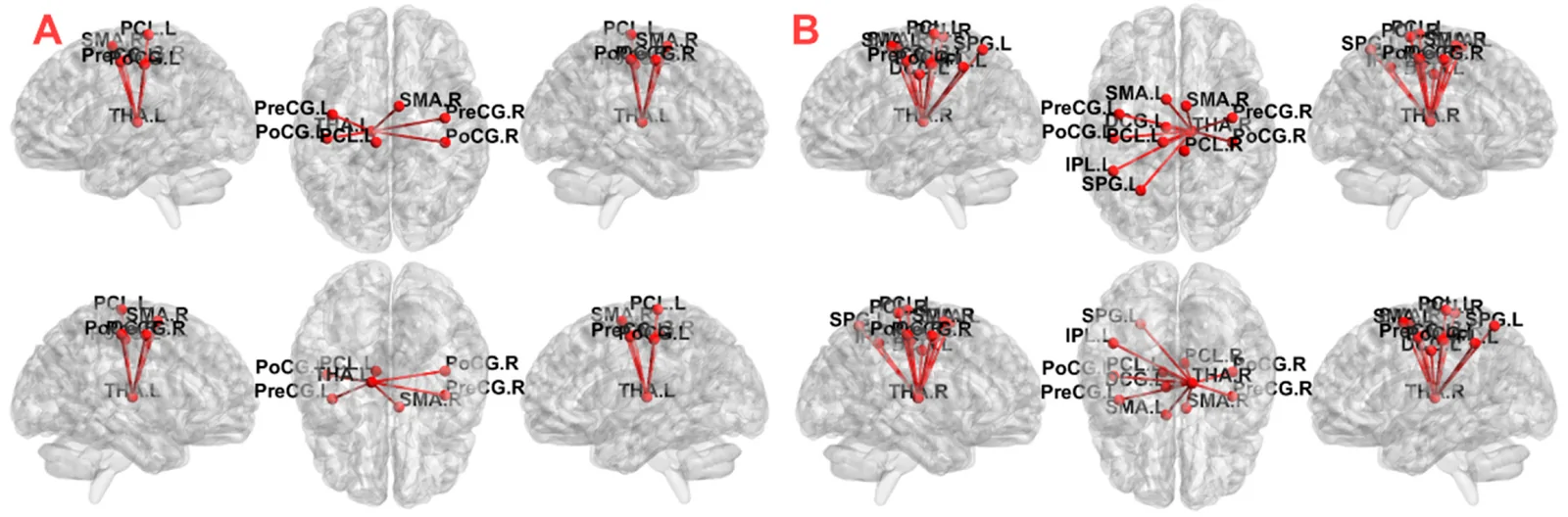
Higher thalamic seed-based functional connectivity in PTs than in HCs. (**A**) With the left thalamus as the seed, three clusters showed higher FC in PTs than in HCs. Cluster 1 involved the left postcentral and precentral gyri, inferior parietal lobule, and supramarginal gyrus (peak MNI coordinates: −45, −18, 57; extent: 366 voxels; peak t = 4.96). Cluster 2 involved the right precentral and postcentral gyri (27, −21, 57; 198 voxels; peak t = 4.43). Cluster 3 involved the bilateral paracentral lobules and supplementary motor areas (6, −21, 66; 139 voxels; peak t = 4.18). (**B**) With the right thalamus as the seed, three clusters showed higher FC in PTs than in HCs. Cluster 1 involved the right postcentral and precentral gyri (54, −15, 27; 306 voxels; peak t = 4.41). Cluster 2 involved the left postcentral, superior parietal, precentral, inferior parietal, and supramarginal regions (−24, −48, 54; 326 voxels; peak t = 4.51). Cluster 3 involved the bilateral paracentral lobules, supplementary motor areas, and middle cingulate cortex (−6, −30, 57; 385 voxels; peak t = 4.75). Red nodes and connections indicate PTs > HCs. Results were corrected using one-tailed GRF correction (voxel-level *p* < 0.001; cluster-level *p* < 0.05), with age and sex included as covariates. Abbreviations: FC, functional connectivity; GRF, Gaussian random field; HCs, healthy controls; MNI, Montreal Neurological Institute; PTs, patients with low back pain.

The third cluster comprised 139 voxels and peaked at MNI coordinates (6, −21, 66), with a peak t value of 4.18. This cluster involved the bilateral paracentral lobules and supplementary motor areas. The spatial distribution of these clusters is shown in Figure 5A and detailed in Supplementary Table S8.

#### 3.5.2. Right Thalamic Seed

Voxel-wise functional connectivity analysis using the right thalamus as the seed identified three increased connectivity clusters in the patient group. The first cluster comprised 306 voxels and peaked at MNI coordinates (54, −15, 27), with a peak t value of 4.41. This cluster involved the right postcentral and precentral gyri.

The second cluster comprised 326 voxels and peaked at MNI coordinates (−24, −48, 54), with a peak t value of 4.51. This cluster involved the left postcentral gyrus, left superior parietal gyrus, left precentral gyrus, left inferior parietal lobule, and left supramarginal gyrus.

The third cluster comprised 385 voxels and peaked at MNI coordinates (−6, −30, 57), with a peak t value of 4.75. This cluster involved the bilateral paracentral lobules, bilateral supplementary motor areas, and bilateral middle cingulate cortex. The spatial distribution of these clusters is shown in Figure 5B and detailed in Supplementary Table S9.

### 3.6. Selection-Dependent Post Hoc Analyses of Extracted Values

Selection-dependent post hoc analyses of the extracted cluster values are presented in Supplementary Table S10. After adjustment for age, sex, and TIV, negative group coefficients were observed for both VBM clusters (Cluster 1: β = −0.064, SE = 0.015, t = −4.21, *p* < 0.001; Cluster 2: β = −0.057, SE = 0.015, t = −3.73, *p* < 0.001).

After adjustment for age and sex, all nine extracted local fMRI measures showed negative group coefficients (β range, −0.866 to −0.129; t range, −5.47 to −4.16; all FDR q < 0.001). Age coefficients were nominally significant for all nine measures (all uncorrected *p* < 0.05), whereas none of the sex coefficients reached *p* < 0.05. In the residual-based analyses, all nine PT−HC differences were negative (β range, −0.523 to −0.078; t range, −3.97 to −3.12; FDR q range, 0.001–0.003).

### 3.7. Clinical Correlation Analyses

Partial correlations between extracted imaging measures and VAS or JOA scores were evaluated in patients (Supplementary Table S11).

No VBM, fALFF, or ReHo association reached nominal *p* < 0.05 (all *p* ≥ 0.05).

**Figure 6:**
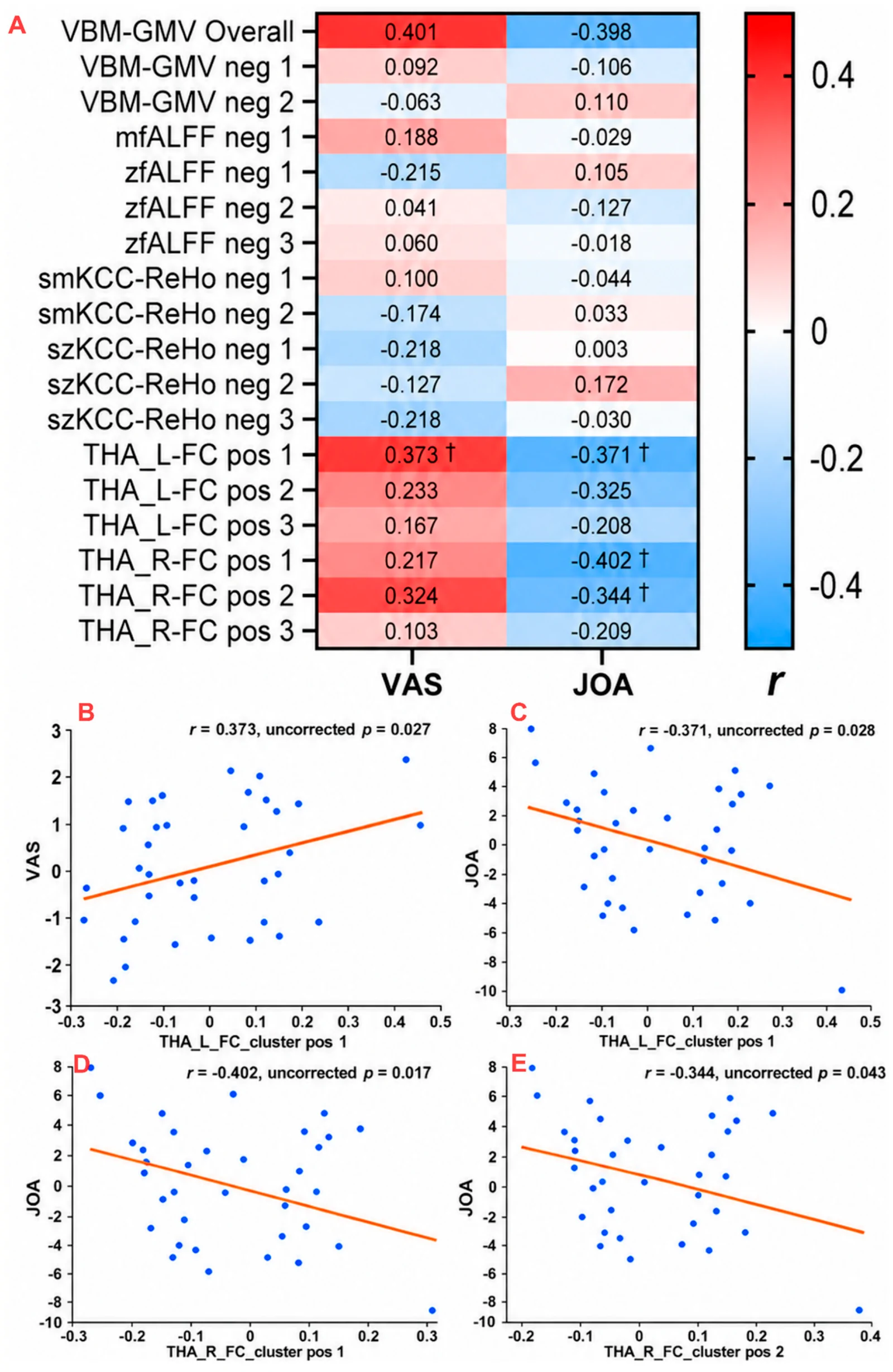
Exploratory partial correlations between cluster-level imaging measures and clinical scores in patients with LBP. (**A**) The heat map displays partial correlation coefficients between imaging measures and VAS or JOA scores. Red and blue indicate positive and negative coefficients, respectively. A dagger (†) indicates an uncorrected exploratory association at *p* < 0.05. Scatter plots show the partial correlations between (**B**) THA_L FC cluster 1 and VAS (partial r = 0.373, uncorrected *p* = 0.027, FDR q = 0.337), (**C**) THA_L FC cluster 1 and JOA (partial r = −0.371, uncorrected *p* = 0.028, FDR q = 0.337), (**D**) THA_R FC cluster 1 and JOA (partial r = −0.402, uncorrected *p* = 0.017, FDR q = 0.337), and (**E**) THA_R FC cluster 2 and JOA (partial r = −0.344, uncorrected *p* = 0.043, FDR q = 0.346). VBM correlations were adjusted for age, sex, and TIV; fMRI correlations were adjusted for age and sex. All displayed *p* values are uncorrected and are provided for exploratory interpretation. None of the displayed associations survived Benjamini–Hochberg FDR correction across the 36 imaging–clinical tests. Abbreviations: FC, functional connectivity; GMV, gray matter volume; JOA, Japanese Orthopaedic Association; mfALFF, mean-scaled fractional amplitude of low-frequency fluctuations; ReHo, regional homogeneity; smKCC-ReHo, smoothed mean-scaled Kendall’s coefficient of concordance-based ReHo; szKCC-ReHo, smoothed z-standardized Kendall’s coefficient of concordance-based ReHo; THA_L, left thalamus; THA_R, right thalamus; TIV, total intracranial volume; VAS, Visual Analog Scale; VBM, voxel-based morphometry; zfALFF, z-standardized fractional amplitude of low-frequency fluctuations. In scatter plots B–E, blue dots represent patient-level residual values after adjustment for age and sex, and orange lines represent the fitted linear trends.

For the left thalamic seed, FC cluster 1 was positively associated with VAS (partial r = 0.373, uncorrected *p* = 0.027) and negatively associated with JOA (partial r = −0.371, uncorrected *p* = 0.028). For the right thalamic seed, FC cluster 1 was negatively associated with JOA (partial r = −0.402, uncorrected *p* = 0.017), as was FC cluster 2 (partial r = −0.344, uncorrected *p* = 0.043). No other FC association reached nominal *p* < 0.05. None of the 36 imaging–clinical associations survived Benjamini–Hochberg FDR correction (all q > 0.05; Supplementary Table S11). The uncorrected exploratory associations are displayed in Figure 6.

## 4. Discussion

### 4.1. Principal Findings

Two complementary features characterized the multimodal results. First, structural volume and local functional measures were lower in the thalamus and related thalamic–hippocampal, orbitofrontal–striatal, temporal, and cerebellar regions. Second, bilateral thalamic connectivity with sensorimotor, parietal, supplementary motor, and middle cingulate regions was higher. Thus, the findings do not indicate a uniform increase or decrease in brain function; rather, they show lower regional measures alongside stronger long-range thalamic coupling. Within the selected clusters, the group coefficients retained their original directions under the alternative covariate treatments. Because the clusters and extracted values were derived from the same group contrasts, these observations are descriptive and do not constitute independent validation or resolve demographic confounding.

The clinical analyses yielded hypothesis-generating, rather than confirmatory, brain-behavior observations. At uncorrected thresholds, left thalamic FC cluster 1 was associated with greater pain and poorer lumbar function, while right thalamic FC clusters 1 and 2 were associated with poorer lumbar function. None survived FDR correction across the 36 tests; these associations are therefore retained as exploratory observations rather than clinically validated markers.

### 4.2. Structural Changes in the Thalamus and Hippocampus

The thalamus links nociceptive and somatosensory input with cortical systems involved in perception, attention, movement, and affect [18,19,20]. The lower gray matter volume observed in the thalamic–hippocampal cluster is compatible with, but does not establish, experience-related plasticity or pain-associated structural change, particularly given the substantial demographic imbalance between groups. Hippocampal involvement is also plausible in this context: pain is shaped by memory, learned context, and stress, not simply by incoming sensory signals. Previous meta-analyses have found gray matter abnormalities in chronic LBP, although their location and direction have not been consistent [21,22]. Variation in clinical phenotype, pain duration, preprocessing, and statistical thresholds may partly explain this heterogeneity.

Cerebellar lobule VIII places the structural finding outside the conventional pain network. Because the cerebellum contributes to sensorimotor integration and adaptation, this change could be related to altered posture or compensatory movement. We did not measure gait, posture, or movement avoidance, however, so this explanation remains speculative.

### 4.3. Decreased Spontaneous Activity in the Thalamus

The thalamic mfALFF and zfALFF maps showed spatial consistency across alternative scaling procedures, but these are different normalizations of the same underlying measure and do not constitute independent replication [14,15]. Lower fALFF should not be read as direct evidence of neuronal loss or reduced nociceptive transmission. It may reflect neural, vascular, physiological, or preprocessing-related contributions that resting-state BOLD data cannot separate.

The orbitofrontal–striatal findings suggest that the functional differences were not confined to sensory pathways. These regions contribute to salience, valuation, affective regulation, and action selection, all of which may influence avoidance and disability in persistent pain. Without psychological or behavioral measurements, though, the present data cannot identify which of these processes is most relevant.

### 4.4. Reduced Local Functional Coherence

ReHo describes local temporal agreement and is therefore complementary to fluctuation amplitude [16]. Its spatial overlap with fALFF in the thalamus indicates that both fluctuation amplitude and local signal coordination differed between the sampled groups. This convergence is descriptive, however, because the measures were derived from the same demographically imbalanced dataset and may share physiological and preprocessing-related influences. Findings in orbitofrontal–striatal and temporal areas point to a wider disturbance of local integration, but the cognitive meaning of these clusters cannot be inferred from anatomy alone.

### 4.5. Increased Thalamo-Sensorimotor and Thalamo-Parietal Connectivity

The connectivity results moved in the opposite direction from the local thalamic measures. Stronger coupling with sensorimotor, parietal, supplementary motor, and mid-cingulate regions could reflect closer monitoring of bodily signals, altered sensorimotor integration, compensatory recruitment, or maladaptive reinforcement. Similar abnormalities in thalamo-sensorimotor connectivity and stronger coupling with somatomotor and frontoparietal networks have been reported in chronic LBP [23,24].

We cannot conclude that the stronger connectivity compensated for the lower local measures, because no subject-level mediation or cross-modal coupling analysis was performed. The middle cingulate and supplementary motor regions are involved in attention, action selection, and pain-related movement control. Their stronger coupling with the thalamus may therefore accompany adaptation to ongoing pain or changes in movement. A longitudinal design would be needed to distinguish adaptation from predisposition or maladaptive plasticity.

### 4.6. Interpretation of the Selection-Dependent Post Hoc Analyses

The age and sex differences between groups remain an important source of uncertainty. The extracted cluster coefficients retained their original directions under the alternative covariate treatments. However, because the clusters were defined using the same group contrasts, these selection-dependent analyses describe only the behavior of the selected estimates and should not be interpreted as evidence of stability or independent confirmation. Age was associated with all nine extracted local fMRI measures, and no statistical adjustment can recreate a prospectively matched sample or resolve the underlying demographic confounding.

### 4.7. Imaging–Clinical Associations

The nominal thalamic-connectivity associations were directionally coherent with greater pain or poorer lumbar function: left thalamic FC cluster 1 correlated positively with VAS and negatively with JOA, and right thalamic FC clusters 1 and 2 correlated negatively with JOA. However, none survived FDR correction across the 36 tests. These findings remain hypothesis-generating and do not establish clinical utility or a brain-behavior marker.

### 4.8. Clinical and Research Implications

Across the sampled groups, the multimodal analyses delineated a distributed pattern involving thalamic structure, local resting-state measures, and thalamo-sensorimotor connectivity. This pattern provides a focused hypothesis for future studies of central contributions to LBP alongside peripheral spinal pathology. However, the present cross-sectional findings do not support individual-level diagnosis, prognosis, phenotyping, or treatment selection.

A useful next step would be to study prospectively matched groups while recording pain duration, psychological state, sleep, medication exposure, and movement behavior. Longitudinal follow-up could then test whether thalamic structure and function change with symptoms or treatment [34,35,36,37]. Independent replication will be essential before the pattern can be considered robust.

### 4.9. Limitations

This retrospective cross-sectional study has several limitations. The groups differed markedly in age and sex, with limited overlap in age distributions; linear adjustment cannot adequately model potentially nonlinear aging effects or substitute for prospective matching. Age was associated with all nine extracted local fMRI measures, so the observed group differences cannot be assigned specifically to LBP. Only 23 patients remained in the VBM analysis after CAT12 quality control, whereas no controls were excluded. Included-versus-excluded comparisons did not reach *p* < 0.05, but this does not establish equivalence, and differential attrition may have introduced selection bias. The pooled patient group included several lumbar disorders, while diagnostic counts, pain duration, medication exposure, and physical-therapy history were not consistently available; the findings therefore cannot be assigned to a single diagnosis.

Multiplicity was controlled within each directional statistical map but not across the two directions or across the complete family of VBM, fALFF, ReHo, and seed-connectivity analyses. The use of two separately corrected one-tailed contrasts without an across-direction adjustment does not control the overall bidirectional Type I error at 0.05. Mean-scaled and z-standardized variants are alternative representations rather than independent confirmation. In addition, the post hoc extracted-value analyses are selection-dependent because the clusters were defined using the same group contrasts; they describe the selected estimates but cannot provide independent validation. Resting-state measures are also sensitive to motion, nuisance regression, global-signal handling, physiological noise, and sample size [38,39,40,41,42]. Friston-24 motion terms and mean white-matter and cerebrospinal-fluid signals were included in nuisance regression, and mean Power FD did not differ significantly between groups. However, volume censoring was not performed and mean FD was not included as a second-level imaging covariate. A nonsignificant average FD comparison cannot exclude transient or spatially structured motion effects. Concurrent cardiac and respiratory waveforms were not recorded, so participant-specific physiological-noise correction was not possible; residual physiological and vascular signals may have contributed to the thalamic findings. Finally, no imaging–clinical association survived FDR correction, and the study did not include individual-level classification, cross-validation, or external validation.

## 5. Conclusions

In this retrospective exploratory study, multimodal MRI identified two complementary between-group features: lower gray matter volume and local functional measures centered on the thalamus and related regions, and higher bilateral thalamic connectivity with sensorimotor, parietal, supplementary motor, and middle cingulate regions. Nominal associations between thalamic connectivity and pain or lumbar function were directionally coherent but did not survive FDR correction and remain hypothesis-generating. Together, these findings delineate a focused thalamus–sensorimotor pattern for future investigation without establishing an LBP-specific or clinically validated marker. Interpretation remains limited by demographic imbalance, clinical heterogeneity, differential VBM attrition, multiplicity across directional and analysis-family tests, selection-dependent extracted-value analyses, and the absence of concurrent physiological recordings. Replication in prospectively age- and sex-matched, clinically stratified cohorts with physiological monitoring is required.

## Figures and Tables

**Table 1:** Participant Characteristics in the fMRI and VBM Datasets.

**(A) fMRI Dataset**			
Variable	HCs, n = 32	PTs, n = 37	*p* value
Age, years	46.84 ± 13.25	67.41 ± 14.07	<0.001
Male, n (%)	8 (25.0%)	19 (51.4%)	0.025
VAS	0.00 ± 0.00	5.95 ± 1.33	<0.001
JOA	29.00 ± 0.00	15.08 ± 4.04	<0.001
Mean Power FD, mm	0.112 (0.085–0.184)	0.155 (0.084–0.225)	0.327
**(B) VBM Dataset**			
Variable	HCs, n = 32	PTs, n = 23	*p* value
Age, years	46.84 ± 13.25	65.30 ± 13.08	<0.001
Male, n (%)	8 (25.0%)	12 (52.2%)	0.039
TIV (ml)	1436.12 ± 110.89	1383.55 ± 111.22	0.089
VAS	0.00 ± 0.00	5.91 ± 1.24	<0.001
JOA	29.00 ± 0.00	15.78 ± 3.74	<0.001

## Data Availability

The clinical and neuroimaging data are not publicly available because they may contain information that could identify participants. De-identified data may be available from the corresponding author upon reasonable request, subject to institutional and ethical approval.

## Supplementary Materials

The following supporting information can be downloaded at: https://www.mdpi.com/article/10.3390/diagnostics16162546/s1, Table S1: head-motion comparison between patients and healthy controls; Table S2: comparison of patients included in and excluded from the VBM analysis; Tables S3–S9. whole-brain VBM, fALFF, ReHo, and thalamic seed-connectivity results; Table S10: selection-dependent post hoc analyses of extracted imaging measures; Table S11: imaging–clinical partial correlations with FDR-adjusted q values.
