# Supplementary material for: Multimodal MRI Characterization of Structural and Resting-State Functional Brain Alterations in Patients with Low Back Pain: A Retrospective Study
Source: Diagnostics (Basel). 2026 Aug 12;16(16):2546. doi: 10.3390/diagnostics16162546 (PMC13511479; doi:10.3390/diagnostics16162546)
Supplement: Supplementary file 1 [file diagnostics-16-02546-s001.zip › diagnostics-4479609-supplementary.pdf]

Supplementary Material

Supplementary Tables

Quality-control analyses are presented first: Table S1 reports the head-motion comparison for the full functional MRI sample, and Table S2 compares patients retained in and excluded from the VBM analysis. Whole-brain structural and resting-state functional MRI findings are presented in Tables S3–S9, including VBM, mfALFF, zfALFF, smKCC-ReHo, szKCC-ReHo, and bilateral thalamic seed-based voxel-wise functional connectivity results. The prefixes “m” and “z” denote mean-scaled and z-standardized measures, respectively. In the cluster identifiers, “pos” denotes higher imaging values in PTs than in HCs (PTs > HCs), whereas “neg” denotes lower values in PTs than in HCs (HCs > PTs). Each directional whole-brain contrast underwent one-tailed Gaussian random field correction at voxel-level $p < 0.001$ and cluster-level $p < 0.05$. The PT > HC and HC > PT contrasts were evaluated separately, without additional correction across directions or analysis families. Selection-dependent post hoc analyses of extracted imaging measures are reported in Table S10. Imaging–clinical partial correlations, including FDR-adjusted q values, are reported in Table S11.

Table S1. Head-motion comparison between patients with low back pain and healthy controls

Motion measure	HCs, n = 32	PTs, n = 37	Effect estimate	p value
Mean Power FD, median (IQR), mm	0.112 (0.085–0.184)	0.155 (0.084–0.225)	Mann–Whitney U = 674.0	0.327
Mean Power FD, mean ± SD, mm	0.148± 0.094	0.178 ± 0.121	PTs−HCs = 0.030 mm (95% CI, −0.022 to 0.081); Welch t = 1.160	0.250
Standardized mean difference	—	—	Cohen’s d = 0.275	—
Age- and sex-adjusted group effect	—	—	β = −0.006 mm; SE = 0.033; t = −0.192; 95% CI, −0.073 to 0.060	0.849

Table S1 note: Power framewise displacement (FD) was calculated across the 230 functional volumes remaining after removal of the first 10 volumes. All 69 participants included in the final functional dataset had passed the prespecified head-motion exclusion criteria of maximum absolute translation ≤3.0 mm and maximum absolute rotation ≤3.0°. Because mean Power FD was non-normally distributed, the Mann–Whitney U test was treated as the primary unadjusted comparison. Mean ± standard deviation and the Welch independent-samples t-test are additionally provided for comparability. The adjusted group effect was estimated using a linear model with mean Power FD as the dependent variable and group, age, and sex as predictors. Positive unadjusted differences and Cohen's d values indicate PTs > HCs; the adjusted β represents the estimated PT−HC difference after controlling for age and sex. Abbreviations: CI, confidence interval; FD, framewise displacement; HCs, healthy controls; IQR, interquartile range; PTs, patients with low back pain; SD, standard deviation; SE, standard error.

Table S2. Comparison of patients included in and excluded from the VBM analysis

Characteristic	Included PT, n=23	Excluded PT, n=14	Effect estimate	p value
CAT12 image quality rating, median (IQR), %	85.93 (83.26–91.45)	76.05 (72.58–78.68)	Not tested—selection criterion	—
CAT12 image quality rating, range, %	80.21–92.98	55.75–79.42	—	—
Age, median (IQR), years	67.0 (64.5–72.5)	74.0 (67.8–77.0)	U=102.0; Hedges' g = −0.388	0.067
Male sex, n (%)	12 (52.2%)	7 (50.0%)	OR=1.091；Fisher's exact test	1.000
VAS, median (IQR)	6.0 (5.0–7.0)	6.0 (5.25–7.0)	U=150.5；Hedges' g=−0.063	0.748

JOA, mean ± SD	15.78 ± 3.74	13.93 ± 4.38	Mean difference = 1.85 (95% CI, −1.05 to 4.75)；Hedges’ g=0.455	0.200
TIV, mean ± SD, mL	1383.55 ± 111.22	1361.32 ± 131.63	Difference = 22.22 mL (95% CI, −64.73 to 109.18)；Hedges’ g=0.182	0.603
Total GM, mean ± SD，mL	518.43 ± 59.87	509.60 ± 46.74	Difference=8.82 mL (95% CI, −27.12 to 44.77)；Hedges’ g=0.156	0.621

Table S2 note: Age and VAS are presented as median (interquartile range) and were compared using the Mann–Whitney U test because of non-normality or ordinal scaling. JOA, TIV, and total GM are presented as mean ± standard deviation and were compared using Welch independent-samples t-tests. Sex was compared using Fisher’s exact test. All tests were two-sided. Hedges’ g is provided as a standardized effect-size estimate; positive values indicate higher values in included than excluded patients. The CAT12 image quality rating was not inferentially tested because the prespecified threshold of ≥80% defined inclusion in the VBM analysis. Comparisons of TIV and total GM should be considered exploratory because estimates derived from images below the quality threshold may be affected by image-quality and segmentation errors. Abbreviations: CI, confidence interval; GM, gray matter; IQR, interquartile range; JOA, Japanese Orthopaedic Association; OR, odds ratio; PTs, patients with low back pain; SD, standard deviation; TIV, total intracranial volume; VAS, Visual Analog Scale; VBM, voxel-based morphometry

Table S3: VBM

Cluster ID	Region	Cluster extent	Peak MNI Coordinate			Peak t	Corrected p
			X	Y	Z		
neg 1		2115	13.5	-34.5	1.5	-4.29	<0.05
	Thalamus_R	1253					
	Thalamus_L	573					
	Hippocampus_L	37					
	Hippocampus_R	19					
neg 2		122	33	-42	-52.5	-3.74	<0.05
	Cerebellum_8_R	122					

Table S4: mfALFF

Cluster ID	Region	Cluster extent	Peak MNI Coordinate			Peak t	Corrected p
			X	Y	Z		
neg 1		35	-6	-12	3	-4.48	<0.05

	Thalamus_R	20					
	Thalamus_L	14					

Table S5: zfALFF

			Peak MNI Coordinate				
Cluster ID	Region	Cluster extent	X	Y	Z	Peak t	Corrected p
neg 1		52	6	15	-3	-4.84	<0.05
	Caudate_R	19					
	Frontal_Sup_Orb_R	8					
	Rectus_R	8					
	Olfactory_R	7					
neg 2		45	-6	-12	3	-4.63	<0.05
	Thalamus_R	25					
	Thalamus_L	17					
neg 3		32	-9	15	0	-4.35	<0.05
	Caudate_L	10					
	Putamen_L	6					
	Rectus_L	6					

Table S6: smKCC-ReHo							
			Peak MNI Coordinate				
Cluster ID	Region	Cluster extent	X	Y	Z	Peak t	Corrected p
neg 1		200	6	15	-6	-4.76	<0.05
	Rectus_R	58					
	Olfactory_R	38					
	Caudate_R	37					
neg 2		143	-21	18	-18	-4.3	<0.05
	Frontal_Inf_Orb_L	44					
	Putamen_L	40					

Table S7: szKCC-ReHo							
			Peak MNI Coordinate				
Cluster ID	Region	Cluster extent	X	Y	Z	Peak t	Corrected p
neg 1		117	60	-63	9	-4.54	<0.05
	Temporal_Inf_R	61					
	Temporal_Mid_R	52					

neg 2		98	9	15	-3	-4.41	<0.05
	Rectus_R	36					
	Caudate_R	19					
neg 3		87	9	-9	3	-6.07	<0.05
	Thalamus_R	51					
	Thalamus_L	30					

Table S8: Voxel-wise FC using the left thalamus as the seed

			Peak MNI Coordinate				
Cluster ID	Region	Cluster extent	X	Y	Z	Peak t	Corrected p
pos 1		366	-45	-18	57	4.96	<0.05
	Postcentral_L	200					
	Precentral_L	132					
	Parietal_Inf_L	14					
	SupraMarginal_L	14					
pos 2		198	27	-21	57	4.43	<0.05
	Precentral_R	114					
	Postcentral_R	72					

pos 3		139	6	-21	66	4.18	<0.05
	Paracentral_Lobule_L	55					
	Supp_Motor_Area_R	46					
	Paracentral_Lobule_R	15					
	Supp_Motor_Area_L	14					

Table S9: Voxel-wise FC using the right thalamus as the seed

Cluster ID	Region	Cluster extent	Peak MNI Coordinate X	Y	Z	Peak t	Corrected p
pos 1		306	54	-15	27	4.41	<0.05
	Postcentral_R	212					
	Precentral_R	63					
pos 2		326	-24	-48	54	4.51	<0.05
	Postcentral_L	133					

	Parietal_Sup_L	85					
	Precentral_L	50					
	Parietal_Inf_L	43					
	SupraMarginal_L	13					
pos 3		385	-6	-30	57	4.75	<0.05
	Paracentral_Lobule_L	132					
	Supp_Motor_Area_R	110					
	Supp_Motor_Area_L	40					
	Paracentral_Lobule_R	30					
	Cingulum_Mid_L	28					
	Cingulum_Mid_R	12					

Table S10. Selection-dependent post hoc analyses of extracted imaging measures

Table S10A: Covariate-adjusted ANCOVA results for extracted VBM measures

VBM measure	β (PT−HC)	SE	t	p	Age p	Sex p	TIV p
Cluster 1	−0.064	0.015	−4.21	**<0.001**	0.752	0.19	0.514
Cluster 2	−0.057	0.015	−3.73	**<0.001**	0.578	0.806	0.038

Table S10B: Covariate-adjusted and residual-based descriptive analyses for extracted fMRI measures

fMRI measure	ANCOVA β (PT−HC)	SE	t	Group p	Group p FDR q	Age p	Sex p	Model R^2	Residual β (PT−HC)	Residual SE	Residual t	Residual p	Residual FDR q
mfALFF cluster 1	−0.129	0.026	−4.96	**<0.001**	**<0.001**	<0.001	0.986	0.301	−0.078	0.021	−3.65	**<0.001**	**0.001**
zfALFF cluster 1	−0.866	0.177	−4.89	**<0.001**	**<0.001**	<0.001	0.138	0.302	−0.523	0.145	−3.61	**<0.001**	**0.001**
zfALFF cluster 2	−0.754	0.143	−5.27	**<0.001**	**<0.001**	<0.001	0.946	0.337	−0.456	0.118	−3.85	**<0.001**	**0.001**
zfALFF cluster 3	−0.829	0.173	−4.80	**<0.001**	**<0.001**	<0.001	0.598	0.337	−0.501	0.141	−3.55	**<0.001**	**0.001**
smKCC-ReHo	−0.287	0.064	−4.47	**<0.001**	**<0.001**	<0.001	0.711	0.268	−0.174	0.052	−3.33	**0.001**	**0.002**

cluster 1													
smKCC-ReHo cluster 2	−0.259	0.062	−4.16	**<0.001**	**<0.001**	<0.001	0.621	0.238	−0.157	0.05	−3.12	**0.003**	**0.003**
szKCC-ReHo cluster 1	−0.618	0.123	−5.03	**<0.001**	**<0.001**	0.014	0.697	0.303	−0.373	0.101	−3.70	**<0.001**	**0.001**
szKCC-ReHo cluster 2	−0.710	0.163	−4.36	**<0.001**	**<0.001**	<0.001	0.986	0.244	−0.429	0.132	−3.26	**0.002**	**0.002**
szKCC-ReHo cluster 3	−0.518	0.095	−5.47	**<0.001**	**<0.001**	<0.001	0.379	0.371	−0.313	0.079	−3.97	**<0.001**	**0.001**

Table S10 note: Cluster values were extracted from regions defined by the same whole-brain group contrasts; therefore, these analyses are selection-dependent and should be interpreted descriptively rather than as independent confirmation. VBM models included group, age, sex, and TIV. Local fMRI models included group, age, and sex. Benjamini–Hochberg FDR correction was applied across the nine local fMRI group tests and separately across the nine residual-based comparisons. VBM p values were not additionally adjusted for multiplicity. Negative β values indicate PTs < HCs. Abbreviations: FDR, false discovery rate; HCs, healthy controls; PTs, patients with low back pain; SE, standard error; TIV, total intracranial volume; VBM, voxel-based morphometry

Table S11. Partial correlations between cluster-level structural and functional imaging measures and clinical indicators in the patient group

Imaging metric	Cluster ID	Clinical scale	N	Partial r	Uncorrected p	FDR q

VBM_GMV	Overall GMV	VAS	23	0.401	0.080	0.370
VBM_GMV	Overall GMV	JOA	23	−0.398	0.082	0.370
VBM_GMV	neg 1	VAS	23	0.092	0.699	0.921
VBM_GMV	neg 1	JOA	23	−0.106	0.655	0.907
VBM_GMV	neg 2	VAS	23	−0.063	0.793	0.921
VBM_GMV	neg 2	JOA	23	0.110	0.644	0.907
mfALFF	neg 1	VAS	37	0.188	0.279	0.615
mfALFF	neg 1	JOA	37	−0.029	0.869	0.921
zfALFF	neg 1	VAS	37	−0.215	0.200	0.554
zfALFF	neg 1	JOA	37	0.105	0.535	0.833
zfALFF	neg 2	VAS	37	0.041	0.811	0.921
zfALFF	neg 2	JOA	37	−0.127	0.455	0.780
zfALFF	neg 3	VAS	37	0.060	0.724	0.921
zfALFF	neg 3	JOA	37	−0.018	0.917	0.943
smKCC-ReHo	neg 1	VAS	37	0.100	0.555	0.833
smKCC-ReHo	neg 1	JOA	37	−0.044	0.795	0.921
smKCC-ReHo	neg 2	VAS	37	−0.174	0.302	0.615
smKCC-ReHo	neg 2	JOA	37	0.033	0.847	0.921
szKCC-ReHo	neg 1	VAS	37	−0.218	0.195	0.554
szKCC-ReHo	neg 1	JOA	37	0.003	0.985	0.985
szKCC-ReHo	neg 2	VAS	37	−0.127	0.453	0.780
szKCC-ReHo	neg 2	JOA	37	0.172	0.307	0.615
szKCC-ReHo	neg 3	VAS	37	−0.218	0.195	0.554
szKCC-ReHo	neg 3	JOA	37	−0.030	0.862	0.921
THA_L seed FC	pos 1	VAS	37	0.373	**0.027**	0.337
THA_L seed FC	pos 1	JOA	37	−0.371	**0.028**	0.337
THA_L seed FC	pos 2	VAS	37	0.233	0.177	0.554

THA_L seed FC	pos 2	JOA	37	−0.325	0.057	0.346
THA_L seed FC	pos 3	VAS	37	0.167	0.336	0.638
THA_L seed FC	pos 3	JOA	37	−0.208	0.231	0.554
THA_R seed FC	pos 1	VAS	37	0.217	0.211	0.554
THA_R seed FC	pos 1	JOA	37	−0.402	**0.017**	0.337
THA_R seed FC	pos 2	VAS	37	0.324	0.058	0.346
THA_R seed FC	pos 2	JOA	37	−0.344	**0.043**	0.346
THA_R seed FC	pos 3	VAS	37	0.103	0.555	0.833
THA_R seed FC	pos 3	JOA	37	−0.209	0.228	0.554

Table S11 note: Partial correlations were adjusted for age, sex, and total intracranial volume for VBM measures and for age and sex for functional MRI measures. All tests were two-sided. Benjamini–Hochberg false-discovery-rate correction was applied jointly across all 36 imaging–clinical tests reported in this table. Uncorrected p values are retained for transparency. Statistical significance after multiplicity correction was defined as FDR q < 0.05. No association survived FDR correction. The prefixes “neg” and “pos” indicate lower and higher imaging values, respectively, in patients compared with healthy controls. Abbreviations: FC, functional connectivity; FDR, false discovery rate; GMV, gray matter volume; JOA, Japanese Orthopaedic Association score; VAS, Visual Analog Scale.
